# The Large Ribosomal Subunit Protein L9 Enables the Growth of EF-P Deficient Cells and Enhances Small Subunit Maturation

**DOI:** 10.1371/journal.pone.0120060

**Published:** 2015-04-16

**Authors:** Anusha Naganathan, Matthew P. Wood, Sean D. Moore

**Affiliations:** 1 The Burnett School of Biomedical Sciences, College of Medicine, The University of Central Florida, Orlando, FL, 32816, United States of America; 2 Seattle Biomed, 307 Westlake Ave N, Suite 500, Seattle, WA, 98109, United States of America; 3 Department of Global Health, University of Washington, 1510 N.E. San Juan Road, Seattle, WA, 98195, United States of America; University of Lethbridge, CANADA

## Abstract

The loss of the large ribosomal protein L9 causes a reduction in translation fidelity by an unknown mechanism. To identify pathways affected by L9, we identified mutants of *E*. *coli* that require L9 for fitness. In a prior study, we characterized L9-dependent mutations in the essential GTPase Der (EngA). Here, we describe a second class of L9-dependent mutations that either compromise or inactivate elongation factor P (EF-P, eIF5A in eukaryotes). Without L9, *Δefp* cells are practically inviable. Cell fractionation studies revealed that, in both the Der and EF-P mutant cases, L9's activity reduces immature 16S rRNA in 30S particles and partially restores the abundance of monosomes. Inspired by these findings, we discovered that L9 also enhances 16S maturation in wild-type cells. Surprisingly, although the amount of immature 16S in 30S particles was found to be elevated in *ΔrplI* cells, the amount in polysomes was low and inversely correlated with the immature 16S abundance. These findings provide an explanation for the observed fitness increases afforded by L9 in these mutants and reveal particular physiological conditions in which L9 becomes critical. Additionally, L9 may affect the partitioning of small subunits containing immature 16S rRNA.

## Introduction

Translation fidelity is controlled on a number of levels; from tRNA aminoacylation [1,2], to mRNA decoding [3,4], to co- or post-translational surveillance [5–7]. Numerous factors have been identified that influence the quality of protein synthesis, which is not surprising considering the complexity and physiological commitment to this essential process. Among these, ribosomal protein L9 (encoded by *rplI*) reduces translation frameshifting, miscoding, and bypassing, but the mechanism for this activity remains a curiosity because L9's location on the ribosome precludes interactions with either the peptidyl transferase center or the decoding site [8–10]. Thus, L9's activity as a fidelity factor indicates that there is an important missing component in existing models of translation.

**Fig 1:**
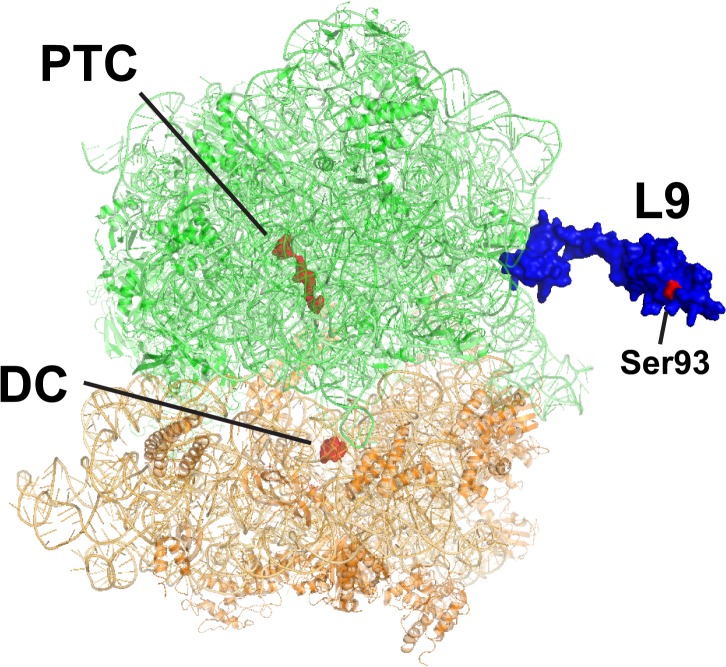
L9 on the ribosome. A crystal structure of the *E*. *coli* ribosome is shown with large subunit RNA and proteins in *green*, small subunit RNA and proteins in *orange*, and L9 in *blue* (PDB entries 3R8S and 4GD1). Residues of the peptidyltransferase center (*PTC*) and decoding center (*DC*) are shown in *red* along with the Ser93 residue in L9 that affects decoding fidelity.

L9 has a highly conserved architecture consisting of two widely spaced globular domains connected by an elongated α-helix (**Fig 1**) [11–13]. A mutation in the C-terminal domain (*hop-1*, Ser93Phe) was isolated as a suppressor of a partially defective translational bypass reporter based on the bacteriophage T4 *gene60* mRNA [14,15]. It was subsequently determined that L9 is required to suppress bypassing, frameshifting, and stop codon "hopping", which suggests that there is a common mechanism behind each of these events [14,16–19]. In addition, it was demonstrated that conserved patches on the globular domains of L9 and the length of the connecting helix affect L9's activity, so the conserved architecture of L9 is also required for its fidelity function [16,18]. Interestingly, despite a remarkable eubacterial conservation, L9 deletion mutants show little growth disadvantage under laboratory conditions [8,19–22].

Mechanistically, it is conceivable that L9 directly influences activities near the E-site, but a direct influence on the peptidyl transferase or decoding centers is hard to reconcile (these are more than 70Å and 90Å away from L9 respectively). Recognizing that the ribosome is a champion of allosteric regulation over large distances, it is possible that L9 imparts a regulatory activity by influencing the decoding center under certain conditions; yet, no evidence for such distortions has been observed in ribosomes lacking L9 using chemical probing or X-ray crystallography [23,24]. However, the structural analyses performed to date did not evaluate ribosomes in the process of frameshifting or bypassing.

As a requisite for establishing a molecular mechanism for L9's function, we implemented a genetic screen to identify physiological situations that impart a requirement for L9. This screen revealed that L9 suppresses growth defects caused by inactivation of the essential ribosome biogenesis factor Der and we recently reported a biochemical characterization of this phenomenon [20]. Here, we report that our screen also revealed mutations in two of the three genes that post-translationally modify elongation factor P (EF-P). Deletion of these genes, or *efp* itself, renders cells highly dependent on L9. The post-translational modification of EF-P enhances its ability to stimulate the synthesis of certain poly-proline motifs when EF-P engages ribosomes between the P and E sites [25–33].

We discovered that both *der* and *efp* mutants exhibit a reduction in 70S pools and show defects in small subunit maturation; each potentially caused by an inability to meet the high ribosomal protein synthesis demand. L9 does not substitute for Der or EF-P activity; rather, L9 improves subunit maturation and partially restores the pools of 70S particles. Taken together, L9's role in enhancing fidelity seems to be most important when free ribosomes become limiting and the demand for high quality protein synthesis is elevated. Surprisingly, an absence of L9 appears to decrease immature 16S abundance in the translation pool.

## Materials and Methods

### Strains and plasmids

Strain TB28 (MG1655, *lacZYIA::frt*) was designated as wild-type for this study [34]. The generation of the *rplI* deletion strain and the unstable plasmid expressing L9 have been described previously [20]. EF-P, EpmA, EpmB, and L9 expression plasmids were cloned into derivatives of pTrc99a [35]. Strain BW30270 (K12 MG1655 *rph+*; CGSC #7925) was used in addition to TB28 for the antibiotic sensitivity studies. A streptomycin-resistant strain, *rpsL25* (CGSC #5522) was used as a control to ensure streptomycin activity was responsible for the dose-dependent toxicity. Δ*rplI::tet* and Δ*prfC::kan* (Keio #JW5873) were transduced into BW30270 for RF3 studies and verified by PCR [20,36]. RF3-influenced frameshift reporter plasmids were previously described (a kind gift from Hani Zaher, Washington University, St. Louis) [5]. Ribosome biogenesis genes and initiation factor clones from the ASKA library were used for complementation tests (kindly provided by Gloria Culver, University of Rochester) [37].

### Screening for L9-dependent mutants

The EF-P related L9-dependednt mutants were recovered from a previous screen [20]. Briefly, an unstable plasmid harboring the only copies of *rplI* and *lacZ* was transformed into an Δ*rplI::tet* TB28 derivative. A chemically-mutated library was then generated and screened for blue colonies (containing cells that needed the unstable plasmid to grow well). The locations of the *epmA* and *epmB* alleles were narrowed using co-transduction mapping using a random-insertion transposon donor library. The mapped regions were then sequenced. Each mapped region had only the reported mutation and *efp*, *epmC*, and *der* were wild-type. Also, Δ*efp* and the previously identified L9-dependent *derT57I* allele are not synthetically lethal with each other [20].

### Ribosome analyses

Cultures were mixed with an equal volume of crushed ice made with HT-10 buffer [20 mM HEPES-Tris, 100 mM K^+^-glutamate, and 10.5 mM magnesium acetate, 0.05 mM EDTA, pH 7.8] prior to harvest. 100X lysates were prepared in HT-10 supplemented with 0.05% Tween-20, 14 mM 2-mercaptoethanol, 20 U/mL RNase inhibitor (Roche), 20 U/mL DNase I (NEB), 0.5 mM CaCl_2_, and 0.1 mg/mL lysozyme, and frozen at -80°C.

Lysates were cleared by centrifugation, normalized by 260 nm absorbance, and aliquots layered onto either 10–40% (polysomes) or 10–30% (subunit) sucrose gradients prepared with supplemented HT-10 using a gradient master (Biocomp) and centrifuged in an SW-41 rotor (Beckman) at 35,000/40,000 rpm (151,000/197,000 x *g*,) for 2.5/4 h at 4°C (polysome/subunit respectively). Profiles were recorded during fractionation with a gradient fractionator (Brandel) or by collecting samples from the bottom (Beckman). Fractions were stored at -80°C. RNA was purified from fractions and analyzed as previously described [38,39]. We evaluated the influence of adding a transpeptidation inhibitor to cultures prior to harvest in an effort to stabilize polysomes as others have reported [29,40]. However, we observed no substantial difference in gradients of lysates pre-treated with 100 μg/mL chloramphenicol. Also, our fractionation method gave comparable polysome profiles using an RNAse deficient strain commonly used for ribosome studies (MRE-600). Primers that amplified the 5’ ends of immature 16S or the 3' region of mature 16S rRNA were used for real-time quantitative PCR [41].

### Targeted L9 Degradation

The L9 degradation system was previously described [20,42]. Briefly, Δ*epmA::kan*, Δ*clpX*, *rplI-tag* strains carrying an inducible protease system (pClpXP) was cultured in exponential phase after induction by diluting cultures into fresh medium containing arabinose. The depletion of L9 was monitored as a function of induction time using Westerns. After approximately 30 minutes of protease induction, L9 levels declined to trace levels. The depletion of L9 was also verified in the harvested samples.

## Results

### L9 is not required for the RF-3 miscoding surveillance system

**Fig 2:**
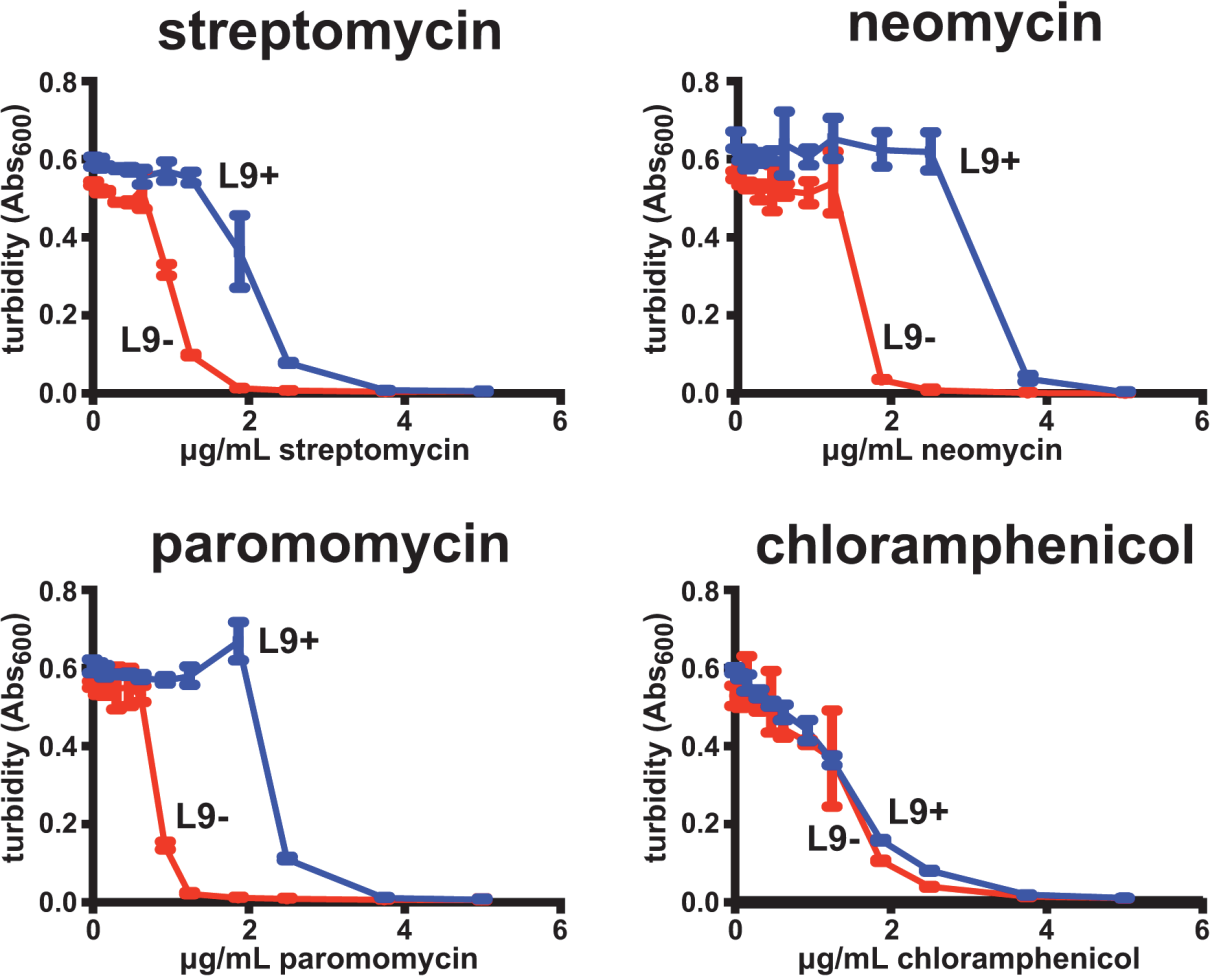
Loss of L9 leads to sensitivity to antibiotics that cause miscoding. A Δ*rplI* (L9-) strains was evaluated for its innate resistance to antibiotics and compared to the isogenic parent (L9+). Consistent with previous reports, the absence of L9 caused only a subtle reduction in growth yield in liquid cultures, but *ΔrplI* colonies are indistinguishable from wild-type. The turbidity of 100 μL cultures grown in a 96-well plate is shown for various concentrations of each drug. The error bars are standard deviations from three experiments.

An outstanding question is whether there is a general reduction of fidelity when L9 is absent, or if such fidelity losses are restricted to situations where frameshifting and bypassing were deliberately enhanced in experimental systems. Therefore, we evaluated L9's influence on growth in the presence of several well-characterized translation inhibitors and discovered that L9 provides a fitness advantage in the presence of antibiotics that promote miscoding (streptomycin, paromomycin, and neomycin), but not an antibiotic that blocks transpeptidation (chloramphenicol, **Fig 2**). Therefore, consistent with reports of decoding fidelity being reduced when L9 is absent in a handful of engineered reporter systems [14,16–19], a loss of L9 appears to reduce translation fidelity in general because these miscoding antibiotics act indiscriminately [3,4].

Recent reports describe a translation quality control system in bacteria that employs release factors 2 and 3 to prematurely terminate the synthesis of proteins in ribosomes with mispaired tRNAs in their P- and E-sites [5,43]. Because mispairing is a requisite for many miscoding events, we considered the possibility that L9 may be involved in regulating this surveillance process, which could readily explain L9's influence on translation fidelity. However, L9 did not influence RF3-mediated miscoding surveillance (**S1 Fig)**. Therefore, the reduction of translation fidelity that occurs in L9’s absence occurs *via* another mechanism.

### Mutations in EF-P modification genes cause L9 dependence

**Fig 3:**
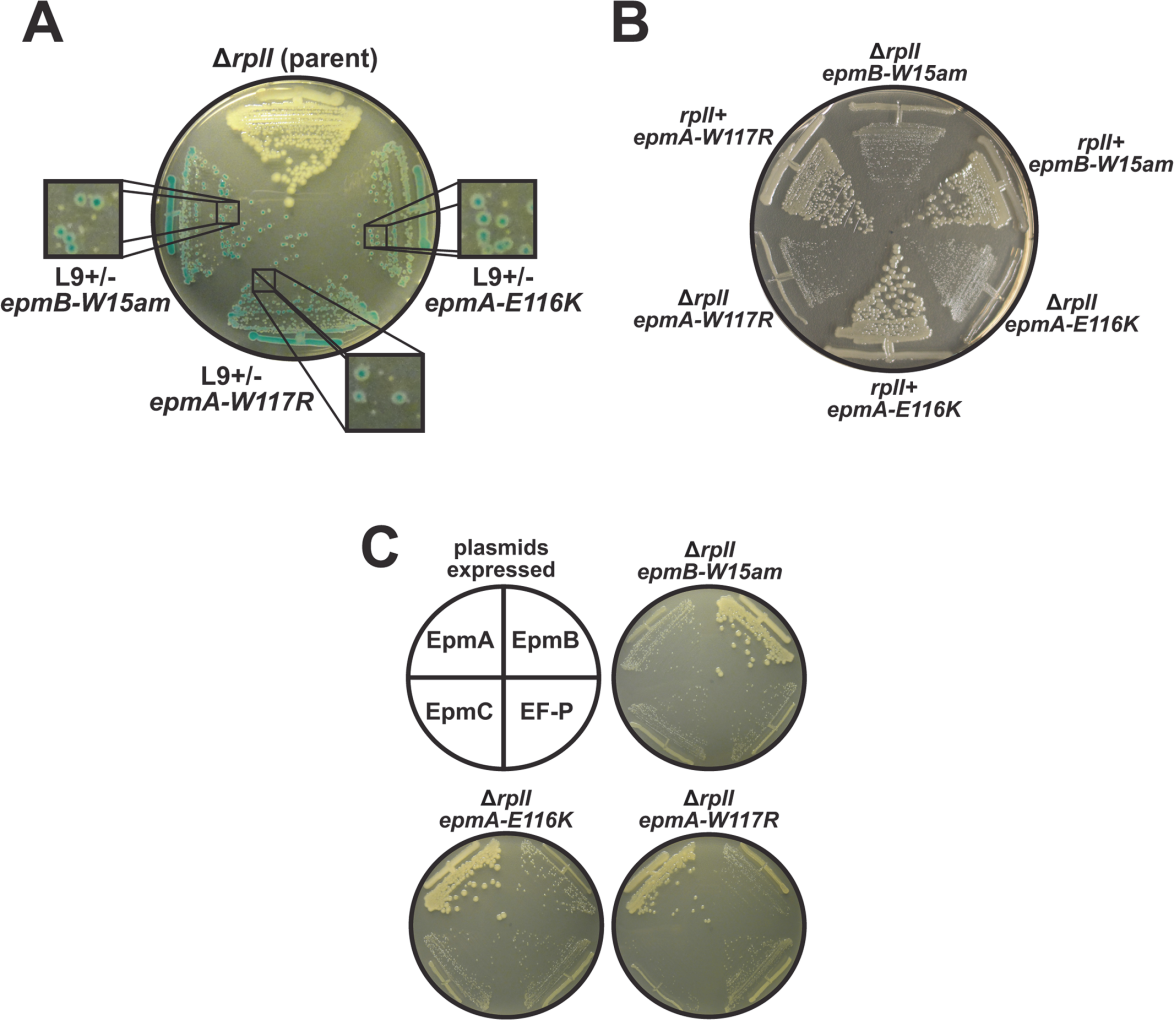
Inactivation of EF-P causes a dependence on L9. A genetic screen revealed mutants that depend on L9 for fitness. (**A**) An X-gal indicator plate streaked with strains harboring a partitioning-defective plasmid that expressed both L9 and LacZ. The Δ*rplI* parent strain grew well without the L9 expression plasmid, which was readily lost upon outgrowth forming white colonies. Three recovered L9-dependent mutants contained defects in genes responsible for post-translationally modifying EF-P. The expanded views highlight the growth differences between colonies seeded from a cell containing the L9 plasmid (blue) and those that were seeded from cells without the plasmid (white). (**B**) Phage transduction was used to restore *rplI* in the chromosome of each mutant using a *cat*-linked locus. The plate shows comparisons of the each mutant with and without L9 support. (**C**) The L9-dependent strains were transformed with plasmids that express wild-type versions of EpmA, EpmB, EpmC, or EF-P. Each mutant was only complemented by its respective wild-type allele. The *epmA-W117R* mutant was only partially complemented by excess wild-type, suggesting this mutant is dominant negative.

Attempts to develop a mechanistic model for L9 function are complicated by a weak L9- phenotype and also by not having an indication of what physiological stresses maintain L9 in nature. To identify such conditions, we used a synthetic lethal screen to identify *E*. *coli* mutants that require L9 for fitness [20]. One class of mutants was found in enzymes responsible for post-translationally modifying EF-P. In many bacteria, EF-P is modified by the addition of hydroxy-β-lysine to a conserved lysine residue and the modification is required for enhancing EF-P's established biological functions [26–31,33,44,45]. One L9-dependent mutant we recovered contained an amber stop codon early in *epmB* (*epmB-W15am*, formerly *yjeK*), whose product converts α-lysine to β-lysine [26,44,46]. In addition, two of the L9-dependent strains contained missense mutations in the predicted active site of EpmA (*epmA-E116K* and *epmA-W117R*, formerly *yjeA* or *poxA*) [27,28], the enzyme responsible for attaching the β-lysine residue to the highly-conserved Lys34 of EF-P. When cured of the L9 support plasmid, each mutant exhibited poor growth (**Fig 3A**).

We confirmed that the reduction in growth rate was indeed caused by a loss of L9 by restoring the *rplI* locus in these mutants (**Fig 3B**). Although the fitness of each mutant was substantially improved when *rplI* was restored, none of the *rplI+* mutants grew as well as wild-type and the *epmA-W117R* mutant displayed the most pronounced slow growth phenotype. In addition, we verified that the mutated EF-P modification genes were responsible for the dependence on L9 by providing wild-type copies of each from a plasmid (**Fig 3C**). Finally, we generated new Δ*rplI* strains containing full deletions of *epmA*, *epmB*, and *efp* ORFs. These strains were extremely sick, only forming very small colonies after 24 h of incubation, so null alleles were likely missed during our screen because of near-lethal phenotypes (**S2 Fig**).

In some bacteria, β-lysyl-EF-P is additionally hydroxylated by EpmC (YfcM) [27]. However, we did not recover mutations in *epmC* and a Δ*rplI*, Δ*epmC* strain grew as well as *epmC+*, which is consistent with reports that this additional modification does not improve EF-P function [29,47]. Taken together, these data indicate that complete inactivation EF-P causes a severe dependence on L9 (they are synthetically lethal) and that our recovered mutants likely maintained a low level of active EF-P because they grow better than Δ*efp* cells [31].

### The conserved L9 architecture is required for improving the growth of Δ*efp* cells

Why is the shape of L9 so highly conserved? Considering L9’s position on the large subunit, it seems that L9 reaches to contact some other factor or a remote region of the ribosome during its function. The necessity of L9 in cells lacking EF-P activity provided a unique platform to interrogate the role of L9's preserved architecture in improving these growth defects. We engineered several variants of L9 expression plasmids and transformed the Δ*rplI*, *epmA*/*epmB/efp* double mutants to test for complementation of the Δ*rplI* allele. We made constructs that expressed each L9 globular domain independently, that expressed the *hop-1* fidelity loss mutant, or that expressed variants with mutations in the connecting helix intended to distort the presentation of the C domain from the surface of the ribosome (a "flexible" linker and a more rigid, "bent" linker) [16,18]. Δ*rplI*, Δ*epmA*/Δ*epmB/*Δ*efp* double mutants were too sick for reproducible microbiological analyses (**S2 Fig**), so we focused on characterizing the point mutants recovered from our screen.

**Fig 4:**
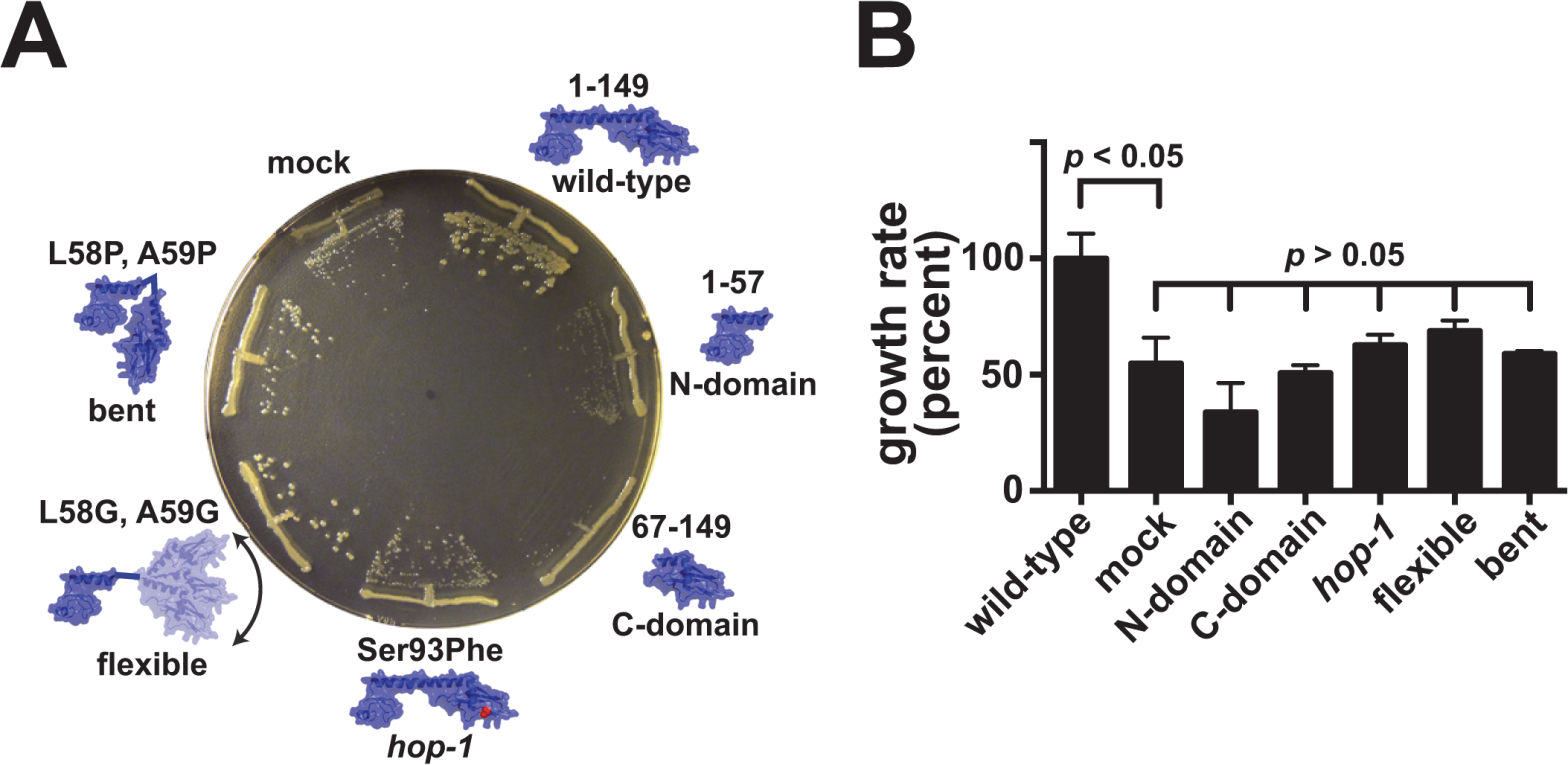
The conserved L9 architecture is required to enhance the mutant growth rates. The L9-dependent mutant Δ*rplI::tet*, *epmB*-*W15am* was transformed with a battery of plasmids that express variants of L9. (**A**) A plate showing the relative colony size differences. (**B**) Liquid culture data of exponential-phase growth rates for the same strains in *panel A*. The N- and C-terminal domains failed to complement and the *hop-1*, *flexible*, and *bent* versions only marginally complemented. Error bars indicate the standard deviations of three independent exponential phase growth rate measurements. Despite discernable colony size differences, the *p-values* from Student's t-tests of the liquid culture growth rate data indicate that the growth rate advantage provided by the even most active the L9 variant (*flexible*) was not substantial.

Each mutant's slow colony growth was suppressed by the L9 variants in the same order: wild-type > flexible > bent > *hop-1* > C domain ≈ N domain ≈ mock (**Fig 4A**). A similar pattern was observed in liquid cultures during exponential growth, but there was more variability; which may have stemmed from the faster division time in liquid culture or the stochastic accumulation of faster-growing escape mutants (**Fig 4B**). In a separate experiment, we established that the *hop-1*, *flexible* and *bent* L9 versions expressed well from these constructs (**S3 Fig**). Because none of the tested L9 variants functioned as well as the full-length wild-type, the positioning and quality of the C domain impacts the ability of L9 to improve the growth defect caused by a reduction of EF-P activity. This observation differs from that of L9-dependent *der* mutants, whose growth rate is fully restored by the N domain alone [20]. The requirement for a wild-type L9 reveals an unprecedented connection between the conserved architecture of L9 and translation elongation efficiency. In the Discussion, we present a tentative model that incorporates the shape of L9 as a key factor.

### Cells with inactive EF-P have reduced monosome levels

We questioned whether L9 affected the ability of EF-P to function or if L9 somehow complemented EF-P activity, perhaps by reducing poly-proline stalling. Cells lacking L9 grow better than Δ*epmA*, Δ*epmB*, and Δ*efp* cells, which indirectly suggests EF-P is functioning normally in this background. Nonetheless, we evaluated EF-P activity in *rplI*+ and *rplI*- cells using a poly-proline translation reporter construct. The expression of the poly-proline reporter was not reduced by an absence of L9 and no influence on EF-P’s ability to rescue poly-proline stalls was observed (**S4 Fig**).

**Fig 5:**
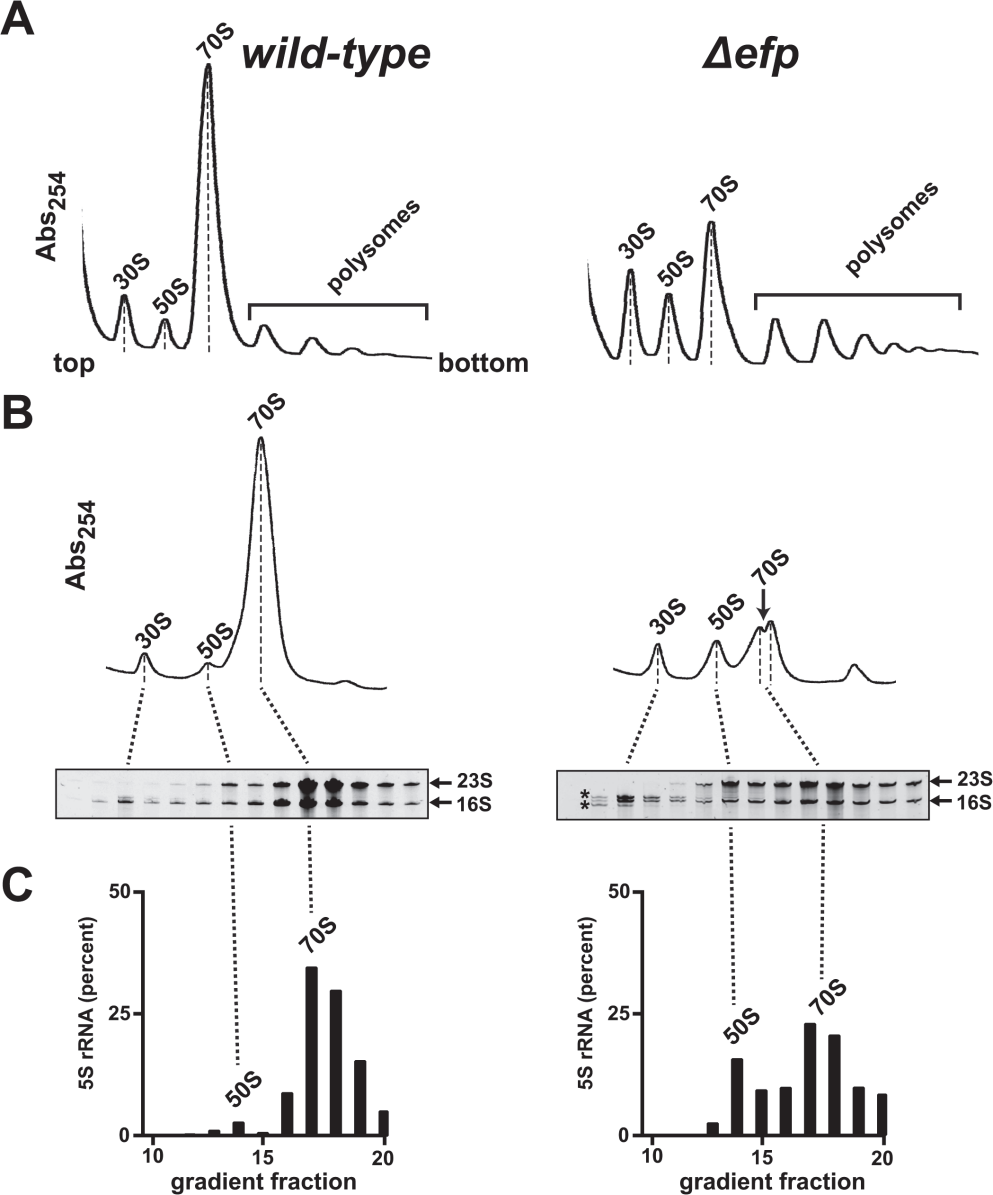
Δ*efp* cells have a deficiency of monosomes. Lysates of *wild-type* and Δ*efp* cultures were normalized by their 260 nm absorbance and resolved in linear sucrose gradients. (**A**) Profiles of each lysate were generated under conditions that resolved polysomes. (**B**) Gradients that resolved subunits from the same lysates in *panel A*. The dashed lines mark the peak centers that were used to calculate the *s*-values of particles and the dotted lines indicate the relative fraction locations. The calculated position of 70S in the Δ*efp* gradient is indicated with an *arrow*. Under each gradient, denaturing gels stained with SYBR green II dye show RNAs purified from the indicated fractions. In the 30S region of the Δ*efp* gradient, RNAs larger and smaller than mature 16S were abundant (*asterisks*). (**C**) In separate gels, the 5S RNA was resolved and quantified from the 50S and 70S peak fractions. The bar graphs show the relative amount of 5S across this region as a percent of the total in those fractions.

Because we discovered a role for L9 in enhancing large subunit maturation in a prior study [20], we set out to characterize ribosomes in the EF-P related mutants to determine if L9 influenced their quality or distribution. The level of monosomes relative to the total in Δ*efp* cells was reduced by ~50%, which is consistent with a prior study of ribosomes from Δ*efp* cells (**Fig 5A**) [29]. When sedimented to better separate subunit peaks, the monosomes from Δ*efp* cells unexpectedly resolved as two peaks with similar 16S/23S ratios (**Fig 5B**). Using the relative spacing of the wild-type peaks as a metric, we calculated apparent *s*-values for these monosome peaks as ~67S and ~72S. Interestingly, we routinely observe shoulders on the monosome peaks derived from wild-type cells, which may be caused by the same monosome variants in different relative abundances. We also observed a comparable reduction of monosomes in Δ*epmA* cells (described below). Consistent with data in a prior report [47], our Δ*epmB::kan* allele exhibited a strong polar effect that concomitantly reduced EF-P levels (**S5 Fig**), so we did not characterize this strain further.

Gel electrophoresis of RNAs purified from the gradient fractions revealed that much of the RNA present in the 30S region of the Δ*efp* gradient appeared to be either immature or fragmented (**Fig 5B**) [48,49]. We did not detect proteins missing from the 30S peak relative to wild-type by Coomassie staining, but small differences may have been masked by an abundance of mature forms. The increase in free 50S subunits in Δ*efp* cells was accompanied by a concomitant increase in the level of 5S rRNA at that peak position (**Fig 5C** and **S6 Fig**). Because the addition of 5S rRNA is a very late stage assembly event [12,50], this finding suggests that large subunit assembly was mostly successful in Δ*efp* cells and that mature large subunits may not have been limiting.

In conjunction with a recent report demonstrating that Δ*efp* cells have reduced levels of several translation-related proteins (including KsgA/RsmA, which regulates 16S rRNA processing) [48,51–53], these data are consistent with a model wherein the loss of EF-P activity caused a deficiency of available ribosomes, possibly through a combination of slowed ribosome recycling, imbalanced protein production, and small subunit maturation defects. In support of this model, our preliminary characterization of second-site suppressors of the Δ*efp* growth defect has revealed that the growth rates correlate directly with the abundance of monosomes (not shown). A more detailed biochemical characterization of both the large and small subunits derived from Δ*efp* cells and their suppressors is planned to determine if the suppression mechanism involves improving the specific activity of subunits.

### Depleting L9 from Δ*epmA* cells exacerbates small subunit maturation defects

Following the observation that Δ*efp* cells have a deficiency of monosomes, we sought to characterize L9's influence on ribosome quality in Δ*efp* cells to gain insight into L9's mechanism of improving the growth rate. Unfortunately, our Δ*rplI*, Δ*epmA*/Δ*epmB*/Δ*efp* double deletion strains were too sick to grow the larger cultures required for ribosome analyses (**S2 Fig**). One strategy to overcome this limitation is to temporarily provide L9 to cells lacking EF-P activity to enhance growth, and then to remove L9 at a convenient time for biochemical analyses. To achieve a conditional removal of L9, we employed a targeted protein degradation system to rapidly deplete L9 in Δ*epmA* cells [20,42]. In this system, a functional L9 variant bearing a C-terminal degradation tag is conditionally degraded by expressing a processive protease (ClpXP) that recognizes the degradation tag. This degradation system rapidly strips and degrades existing L9 from ribosomes and decreases the steady-state levels substantially [20]. We established that the tagged L9 versions support the growth of EF-P related mutants as well as untagged L9 (not shown).

We elected to characterize the effects of L9 depletion in Δ*epmA* cells (as opposed to Δ*epmB* or Δ*efp*) because they were the healthiest when supported by L9 and they did not display additional growth rate reductions when ClpX was absent (**S2 Fig** and not shown). After allowing the L9-supported Δ*epmA* culture to enter exponential phase, the degradation system was activated to degrade L9-deg and the cultures were grown for an additional 60 minutes prior to harvesting. Using Western blots that monitored L9 levels, L9-deg levels declined over a period of ~15–30 minutes to a steady state trace level (not shown). Therefore, this harvest time represents ~30 minutes of growth with thorough L9 depletion. A parallel control culture contained an L9 variant with a stable tag (L9-cont).

**Fig 6:**
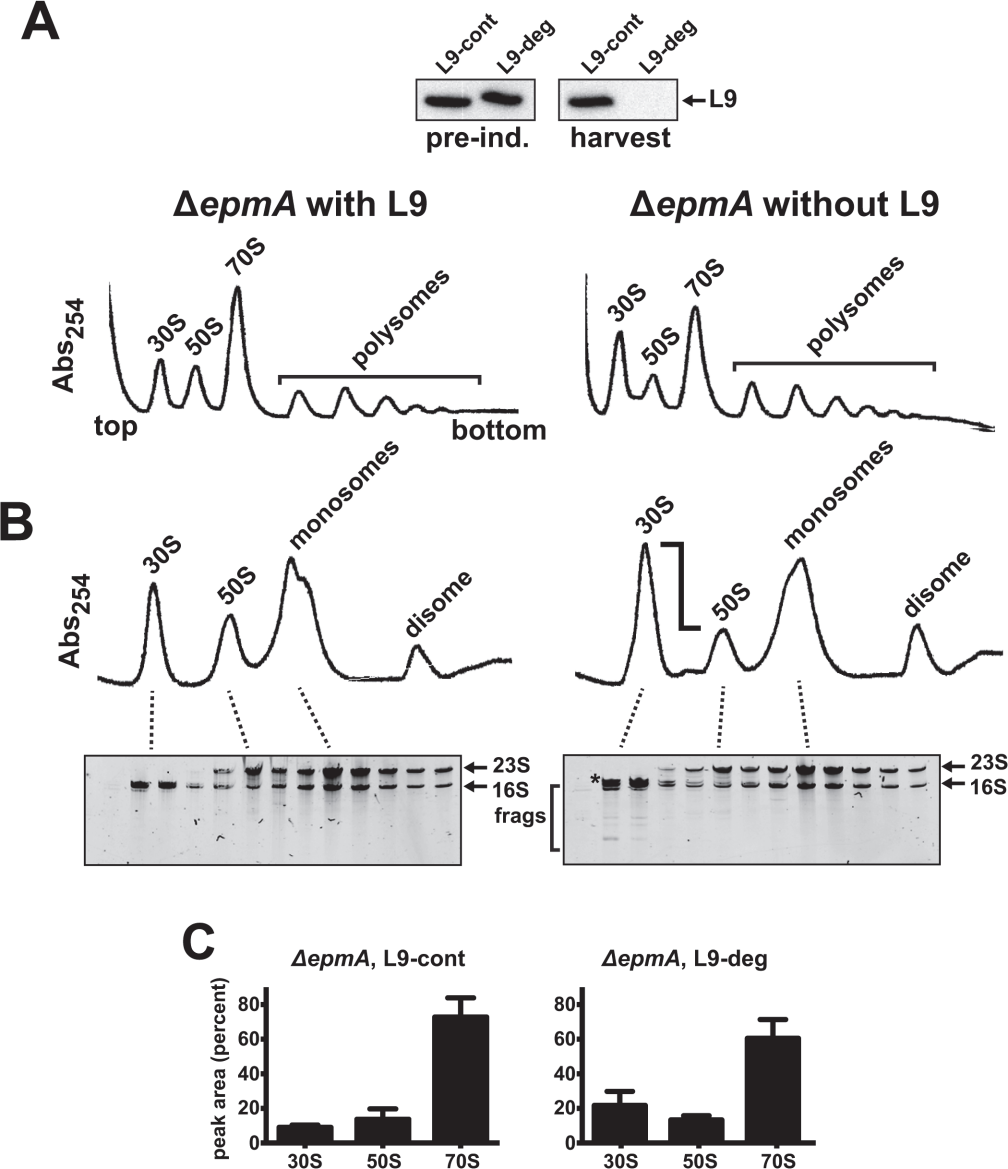
Depleting L9 from Δ*empA* cells exacerbates small subunit defects. Cultures of Δ*epmA* cells expressing L9 with either a control or degradation tag were grown to early exponential phase prior to the expression of ClpXP protease to degrade *L9-deg*. Lysates were then prepared for cell fractionation studies. (**A**) A Western blot showing L9 levels before induction of the degradation system (*pre ind*.) and at the time of *harvest*. L9 was thoroughly depleted in the L9-deg culture, but not in the L9-cont culture (top panel). With L9 support (cells with the stable L9-cont), the ribosome profiles were reminiscent of those from *rplI+* Δ*efp* cells, displaying a reduction in monosomes (left panel). In the culture depleted of L9, the monosome pool was further reduced and 30S particles hyper-accumulated. (**B**) Sucrose gradients for each lysate are shown with gels of purified RNAs. The monosomes resolved as two peaks and the depletion of L9 altered their relative abundances. In addition, 30S particles became more abundant, additional immature 16S rRNA accumulated (*asterisk*), and RNA fragmentation was evident (*frags*). (**C**) The abundance of particles in Δ*epmA* cells with L9 support (L9-cont) or with L9 depleted (L9-deg) was quantified from three experiments. Both monosome peaks were integrated together and considered as "70S" for these comparisons.

The ribosome quality of the L9-supported (L9-cont) Δ*epmA* culture was reminiscent of a Δ*efp* profile, with a heterogeneous monosome peak (**Fig 6**). The depletion of L9 in this mutant caused an additional reduction in monosomes and an over-accumulation of 30S particles. Interestingly, the relative abundance of the two monosome peaks changed when L9 was depleted, suggesting that these forms are differentially affected by L9 activity. Peak areas from gradients of three independent experiments were quantified and we determined that the relative amount of 30S particles approximately doubled when L9 was depleted whereas the level of 50S was essentially unchanged (**Fig 6C**). As with the Δ*efp* cells, the 30S peak had an abundance of particles containing immature 16S rRNAs (**Fig 6B**). Upon L9 depletion, the abundance of this immature RNA increased and additional RNA fragmentation became evident (**Fig 6B**). We also evaluated ribosome quality from the same culture at later harvest time (additional 60 min) and the qualitative findings were the same (not shown). Thus, L9's ability to improve the growth of Δ*epmA* is correlated with improved maturation of small subunits and a moderate increase in monosome abundance.

### L9 improves large subunit quality in a Der mutant and improves 16S maturation

**Fig 7:**
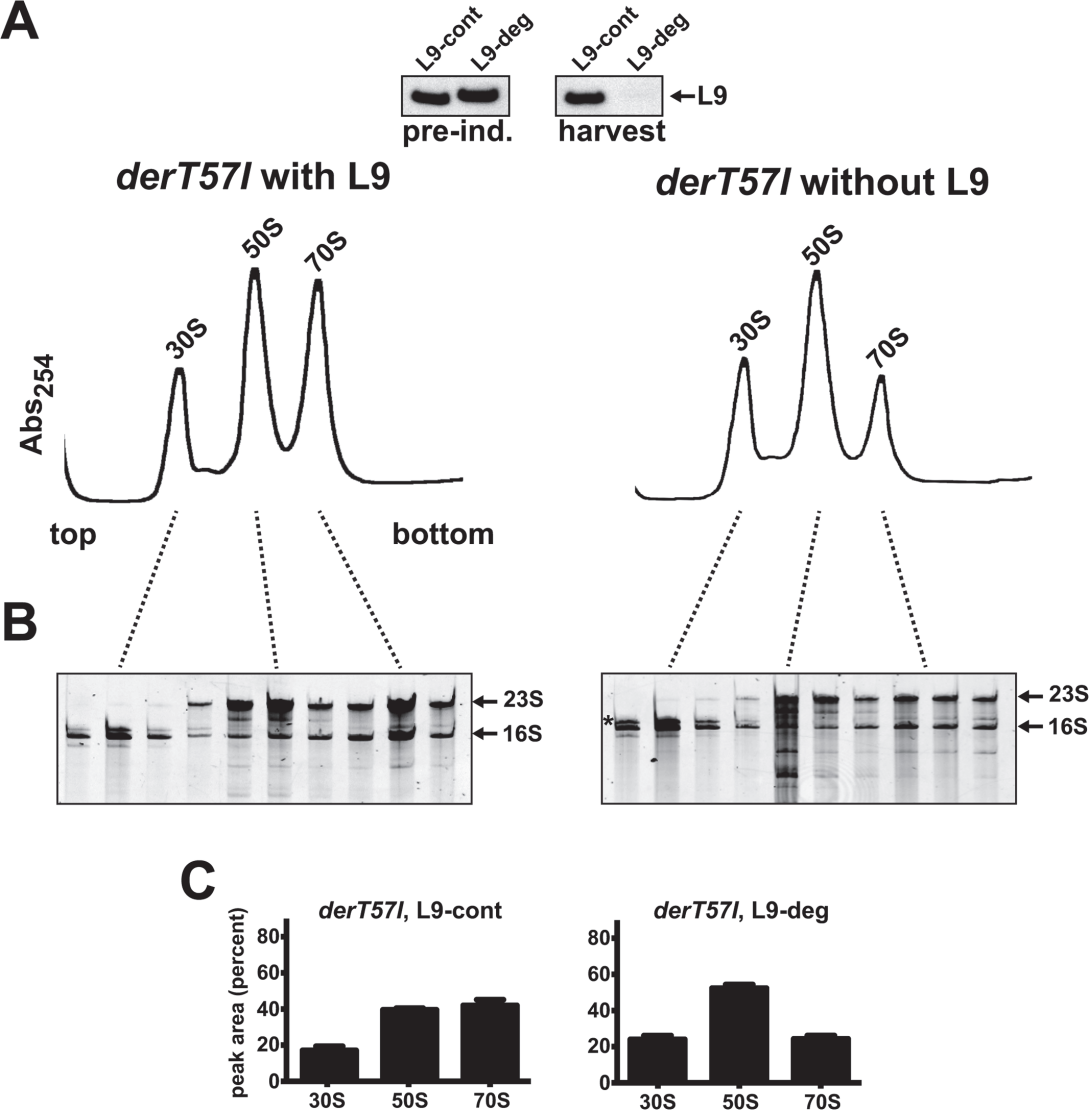
Depleting L9 from *derT757I* cells also exacerbates a monosome deficiency. Cultures of *derT57I* cells with L9-cont or L9-deg were grown to exponential phase prior to depleting *L9-deg*. (**A**) A Western blot evaluated L9 depletion (top). With L9 support (*L9-cont*), the level of 70S particles was substantially reduced compared to *der+* cells and subunit material accumulated between the 30S and 50S peaks. L9 depletion further reduced the 70S peak. (**B**) RNA gels revealed that *derT57I* caused an increase in immature 16S rRNA (*asterisk*) and substantial 23S RNA fragmentation. Depleting L9 exacerbated both of these defects. (**C**) Particle abundances in *derT57I* cells with and without L9 support quantified from three experiments.

In a previous report, we showed that mutations in Der also cause an L9-dependence that is satisfied solely by the N-terminal ribosome-binding domain [20]. In that study, we implemented the targeted degradation system to deplete L9 in a *derT57I* mutant (the more severe of the two recovered *der* mutants), but we did not evaluate the quality of ribosomes under those conditions. Following our findings in EF-P related mutants, we revisited this *der* mutant to determine if L9 also influences ribosome subunit quality in this background. In cells supported with L9, *derT57I* exhibited a stark deficiency of monosomes and increased 30S and 50S particle abundances, consistent with reports of ribosome assembly defects upon long-term Der depletion (Fig 7A and 7C) [54–57]. However, unlike EF-P deficient cells, the monosome peak appeared homogeneous.

Depleting L9 from the *derT57I* cells caused an additional reduction in monosomes and an accumulation of incompletely matured 30S, similar to the case of Δ*epmA* (Fig 7A and 7C). However, unlike the L9 depletion study in Δ*epmA* cells, these changes in particle abundances were concomitant with a severe fragmentation of 23S RNA in the 50S peak (**Fig 7B**). This finding is consistent with a role for L9 in stabilizing the large subunit during late stage assembly when Der activity is limiting. Curiously, in conjunction with these changes, immature 16S rRNA also hyper-accumulated in *derT57I* 30S particles.

### Δ***rplI* cells accumulate immature 16S rRNA in their 30S subunits, but not in their polysomes**

The preceding studies suggested that L9's activity influenced small subunit maturation in two cases in which the monosome pool was compromised for different reasons. Recent reports suggest that when small subunits with immature 16S rRNA enter the translation pool, decoding fidelity is reduced [49,58–62]. These findings raised the exciting possibility that L9's established role as a fidelity factor may stem from this same mechanism. Therefore, we examined the quality and distribution of small subunit RNAs in otherwise wild-type Δ*rplI* cells.

**Fig 8:**
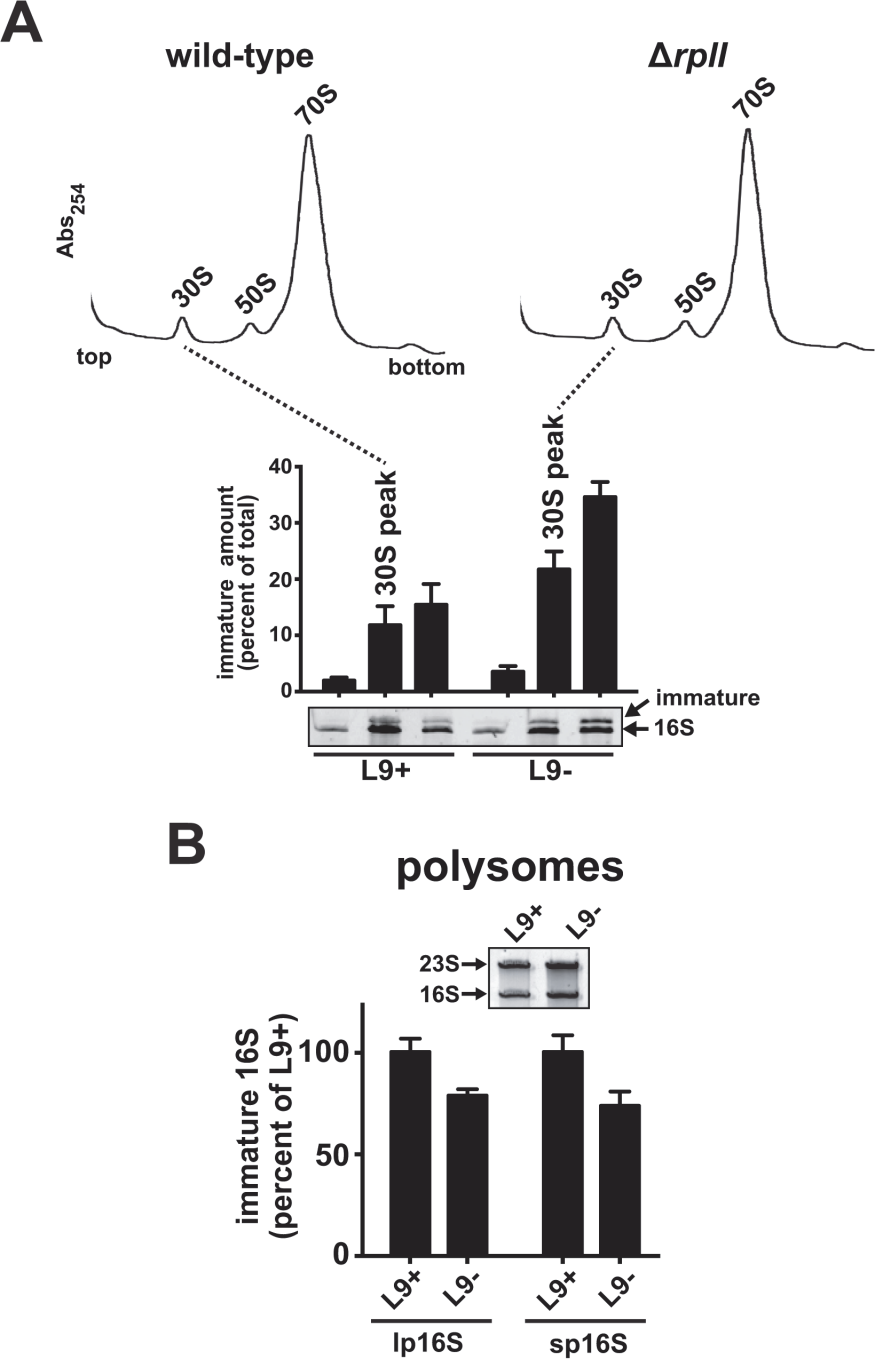
Immature 16S rRNA is overabundant in Δ*rplI* 30S particles, but reduced in polysomes. Lysates were prepared from wild-type and Δ*rplI* cells and resolved using sucrose gradients. (**A**) 30S peak RNAs from each gradient were recovered by fractionating from the top-down, resolved in denaturing gels and stained with SYBR green II prior to densitometry. The quantified immature 16S from two experimental repeats and three measurement repeats is shown as a bar chart with the abundance reported for each of the three peak fractions. (**B**) Polysome RNA samples were collected using bottom-up fractionation. The inset shows RNA from the recovered polysomes, the immature precursor is not evident. The bar chart shows the abundance of immature lp16S (additional 115 5' nucleotides) and sp16S (additional 66 5' nucleotides) relative to total 16S determined by RT-qPCR. The amount of immature 16S is lower in the polysomes from Δ*rplI* cells despite being overabundant in the 30S particles. Error bars are standard deviations from two experimental repeats and three quantifications each.

Although an absence of L9 did not affect the abundance or distribution of ribosome particles in sucrose gradients (**S7 Fig**), we discovered that the 30S particles from Δ*rplI* cells contained approximately twice as much immature 16S rRNA when compared to wild-type (**Fig 8A**). Immature 16S rRNA in 70S and polysomes was undetectable using stained gels, and it was previously established that the amount of precursor in 70S particles is low [49]. We felt it was important to quantify 16S precursors in polysomes directly because 70S particles in sucrose gradients are typically a mixture of monosomes (engaged with tRNAs and mRNA) and contrived species formed by excessive magnesium driving idle subunits together, which do not necessarily reflect the competent translation pool. Therefore, we developed a highly-sensitive RT-qPCR assay to detect established 16S precursors in polysomes [41,49].

In preliminary experiments, we detected higher levels of precursor 16S in polysomes from Δ*rplI* cells. However, because the qPCR method is very sensitive, we determined that this apparent elevation was due to small subunit contamination from top-down fractionations (**S8 Fig**). Therefore, we fractionated separate gradients from the bottom-up for this experiment and prepared RNA for qPCR from those pooled polysome fractions.

Normalized RNA samples were subjected to RT-qPCR reactions that detected total 16S, or the "short precursor" (sp16S) or "long precursor" (lp16S) versions of the immature 5' end [49]. In wild-type polysomes, we detected each immature form (**Fig 8B**). Highly differential detection efficiencies for each species prevented us from establishing precursor to mature ratios using this technique. Surprisingly, the amount of immature 16S was *lower* in ΔL9 polysomes (~75% of wild-type). We also observed elevated immature 16S rRNA in 30S particles, but reduced in polysomes, after activation of the L9 degradation system in otherwise wild-type cells (**S8 Fig**). While this perplexing finding suggests that L9 may be part of a regulatory mechanism that controls the presence or distribution of immature subunits in the translation pool, an abundance of immature 16S rRNA in translating ribosomes is not likely to be the molecular cause of fidelity loss in *rplI* mutants.

## Discussion

We have discovered that L9 and EF-P are synthetically lethal with each other, a phenomenon that apparently stems from L9 partially restoring the pool of monosomes and improving small subunit maturation, which may be linked phenomena. It is important to emphasize that without a full-length, wild-type L9, Δ*efp* cells are nearly inviable, which places special emphasis on the relationship between L9's conserved structure and this particular translation factor. Because L9 also restores the monosome pool in a *der* mutant, it appears that that L9 becomes important when monosomes become limiting. Curiously, there are many plausible ways to disrupt ribosome biogenesis, but all six independently recovered L9-dependent mutations are related either to Der or EF-P.

Although several general models related to translation fidelity could explain the observed L9-related physiological changes, an examination of the molecular contacts and function of L9 during translation will be needed to tease apart a detailed mechanism. Δ*rplI* cells grow nearly as well as wild-type and we determined that L9 is not required for EF-P's function. Therefore, L9's activity is not likely to be specific to the expression of proteins that contain EF-P dependent motifs. Nonetheless, we made an effort to determine if L9 affected the distribution of EF-P in sucrose gradients to see if there was a change in the abundance of EF-P engaged ribosomes. Unfortunately, we were only able to detect unassociated EF-P in the tops of sucrose gradients, not in polysomes (**S5 Fig**). This observation is consistent with a report that EF-P acts quickly to resolve translation problems [31].

When EF-P activity becomes limiting or absent, the lifetime of stalled ribosomes is expected to increase and allow for other molecular events to influence the translation of those messages (such as altering mRNA degradation, crowding ribosomes, or activating toxin systems). As a corollary, the rRNA found in the 30S peaks from Δ*efp* cells is reminiscent of the rRNAs generated by activation of the MazF toxin [59,63,64], so we are inspired to characterize the influence of this RNase on L9-related events in future work. Also, we currently do now know the nature of the two forms of monosome that appear in Δ*efp* cells, but based on an early report by another group [65], we suspect the slower-migrating particles are complexes of small and large subunits associated by the excess magnesium and that the faster-migrating form is a monosome (engaged with mRNA).

**Fig 9:**
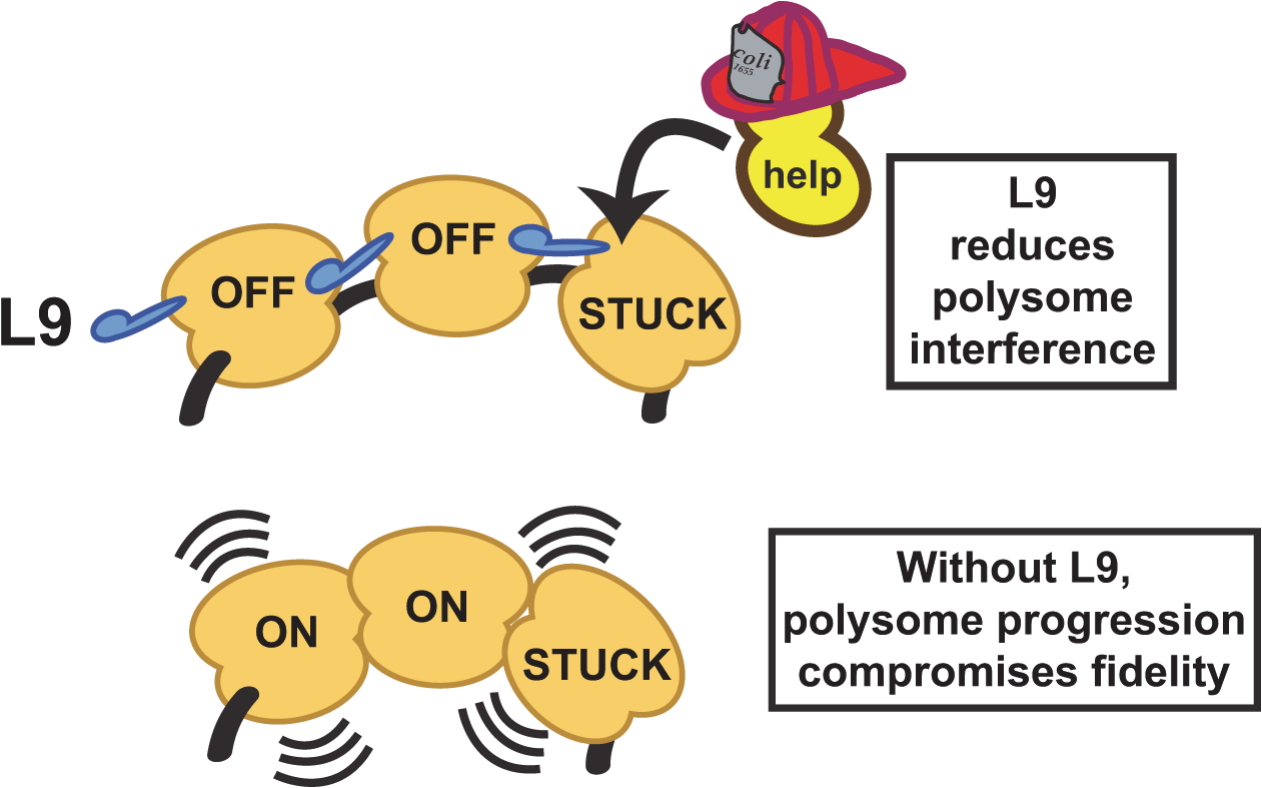
A model for L9 function. L9 may enhance the fidelity of ribosomes in the context of polysomes. In this model, L9's C domain temporarily slows the forward movement of ribosomes that trail transiently stalled ribosomes. Without L9, the uncontrolled forward thrust of trailing ribosomes can compromise fidelity.

Translation pauses also occur during miscoding events, programmed stalling, and at internal Shine-Dalgarno sequences [8,66]. A recent report shows that *E*. *coli* polysomes condense and form ordered arrays when stalling is pronounced [67]. Moreover, a different report revealed that the requirement for EF-P is directly related to the loading efficiency and ribosome occupancy on mRNAs [68]. From these and other observations, we postulate a simple mechanism for how L9 could affect translation fidelity under a variety of different circumstances that involve transient stalling. The model places L9 as a regulator that temporarily shuts down trailing ribosomes (**Fig 9**). In doing so, L9 could reduce forward (plus) frameshifting by protecting stalled/mispaired ribosomes from the collective thrust of trailing ribosomes. In a similar manner, L9 could reduce reverse (minus) frameshifting when ribosomes run into obstructions while they are engaged with “slippery” sequences. In support of this aspect of the model, L9 forms a bridge between adjacent ribosomes in crystal structures and occludes the binding of factors at adjacent GTPase-activating centers [21,24]. Likewise, the GTPase-activating center of a trailing ribosome would be occluded by L9 if stalled polysomes condense to a similar state. This model also nicely supports the observation that Δ*rplI* cells have a reduced level of immature 16S in their polysomes: L9 would help to protect and maintain these ribosomes as they struggle along the mRNAs, akin to ribosomes being affected by aminoglycosides.

Such a mechanism may also require the C terminus of L9 to be presented in different positions from the ribosome body depending on the status of the stall, which would explain the observed dependence on the rigidity and length of the connecting helix. Interestingly, in both EF-P and Der engaged ribosomes, the L1 stalk is rotated over the E-site and the L9 binding site at the base of the L1 stalk concomitantly repositioned [25, 69].

In the case of Der, there is evidence that the large subunits produced in Der's absence are structurally compromised (hyper-sensitive to magnesium depletion) [56]. Although the same model for L9 function would allow enhanced protection of stalled ribosomes with unstable large subunits, only the ribosome-binding N domain of L9 is required to alleviate the Der-related defects. In addition, L9 is reported to be one of a few proteins that dissociates from large subunits produced in Der's absence when magnesium is depleted *in vitro*, so it seems more likely that L9 aids in stabilizing a mature 50S conformation and partially compensates for a slow maturation step. Further evidence for a subunit stabilization model is provided by a recent publication showing the extensive contacts between Der and a contorted large subunit [69].

Immature small subunits over accumulate when L9 is absent and this increase is correlated with L9’s ability to enhance growth of the *der* and *efp* mutants. Others have reported that small subunit maturation is also impaired by sub-lethal doses of aminoglycosides [62,70]. By rapidly depleting L9 in the *derT57I* mutant, we discovered that immature 16S over accumulation is a downstream consequence of a large subunit defect. There are several possible explanations for a delay in small subunit maturation, including a deficiency in the quality or production of small subunit proteins or processing RNases. However, genes associated with translation fidelity and the stringent response are wild-type in fast-growing Δ*efp* escape mutants we have analyzed (**S1 Table**). Continuing this effort, we recently sequenced the genomes of several fast-growing Δ*efp* and Δ*rplI*/Δ*efp* escape mutants and have tentatively identified the mutations responsible. The identified mutations are not in ribosomal genes or in any genes associated with ribosome production in the literature.

The RNA processing of the 16S 5’ end is regulated by the methylation activity of KsgA, one of several proteins found to be deficient in cells lacking EF-P activity [47,51,52]. However, providing additional copies of *ksgA* and other biogenesis genes on plasmids did not enhance the growth of Δ*efp* mutants (**S2 Table**). Nonetheless, a late stage in small subunit production is a logical place for regulating the flow of small subunits into the translation pool from a heterogeneous assembly pathway, a model supported by other observations [71–74]. Although we did not characterize the 3' end of the immature 16S rRNA found in this study, the presence of the long precursor 5' end is evidence it may be 17S rRNA serving as an assembly scaffold [74]. Because we observed immature 16S hyper-accumulation in three different genetic backgrounds, we cautiously suggest that elevated immature 16S rRNA in small subunits is an effect, rather than a cause, of some associated physiologies.

The polysome regulation model we presented above takes into account the shape of L9 and its location on the large subunit. However, other mechanistic models for L9 function need to be considered in light of the observed accumulation and repartitioning of small subunits with immature 16S rRNA. For example, L9 may act to orchestrate stoichiometric maturation/activation of subunits to reduce wasteful idling and subunit turnover in the absence of partners. Alternatively, L9 may be more directly involved in the maturation process, perhaps by recruiting or repelling certain RNases. Such a regulation would be optimal at late stages in assembly, after the established feedback checkpoints governing ribosomal RNA and protein production have been passed.

## Supporting Information

S1 Fig L9 is not required for RF3-mediated miscoding surveillance. Test strains were transformed with plasmids that express reporters based on the well-characterized frameshift sequence found in *prfB* (kind gifts from Hani Zaher, Washington University in St. Louis). The constructs are described in reference [5]. In wild-type cells, frameshifting events are detected in the ribosome and the products are prematurely released through the activity of release factor 3 (RF3). Both non-frameshifted and prematurely released products migrate at the *conventional* position in Western blots. Frameshifted products are longer because they read through an otherwise in-frame stop codon adjacent to the frameshift motif. The top panel is an *anti*-His_6_ Western that detected all reporter products. The bottom panel is an *anti*-cMyc Western that only detected frameshifted material. The "*strong*" reporter contained a bona fide *prfC* sequence that promoted a high level of frameshifting. The "*weak*" reporter had alterations that reduced the level of frameshifting. The frameshifted material in Δ*rplI* cells was not statistically different than that observed in wild-type cells when separate Westerns were used to more accurately quantify the ratios of the two products using dilution series.
(EPS)

S2 Fig Removing L9 from Δ*efp*, Δ*epmA*, or Δ*epmB* cells causes a severe growth defect. Strains containing deletions of *efp*, *epmA*, and *epmB* were subsequently transduced to Δ*rplI*. The plate shows a comparison of the respective pairs. EF-P is partially functional without its modification, which explains the better growth of the Δ*epmA* and Δ*epmB* strains. Also, the reduced growth of Δ*epmB* compared to Δ*epmA* is likely caused by a concomitant reduction of EF-P levels (**S5 Fig**).
(EPS)

S3 Fig Expression of L9 variants from plasmids. Transformed strains were induced to express L9 variants and total protein was analyzed using SDS-PAGE. Each full-length version and the C domain expressed to high levels. The N domain construct did not accumulate to high levels, but was able to fully complement *der* mutants.
(EPS)

S4 Fig EF-P does not require L9 to function. Reporter constructs were used to evaluate EF-P function in different hosts. (**A**) A schematic of the reporters used. Control (AST) and experimental (PPP) sequences were appended to a GFP-based expression plasmid. After these motifs, there were three additional codons before the stop codon. (**B**) Western blot of whole cell lysates that were induced to express the reporter proteins (*arrows*). The antibody detected the His_6_ epitope that preceded the evaluation motif. Relative to the control, the poly-proline construct was expressed poorly in each case. In the Δ*efp* host, the expression of both constructs was increased and a doublet was apparent in the Western. The bottom panel is the same region from a Coomassie stained gel of the samples. The differences in expression levels correlated directly with the mRNA levels of the reporter mRNAs, which were quantified using RT-qPCR on total RNA extracted from separate aliquots of the same cultures at the time of harvest (not shown). (**C**) Mass analyses of purified reporter proteins. A separate aliquot of each culture was processed under denaturing conditions using nickel affinity to purify the reporter proteins from each culture. Each sample was subjected to MALDI-TOF and the counts are reported as *m*/*z*. The calculated masses of the full-length control and poly-proline reporters are 28,274 Da and 28,304 Da respectively. Full-length reporter protein was detected in abundance in each of the control samples and also in the wild-type and Δ*rplI* poly-proline samples. Translation of full-length poly-proline reporter was compromised in the Δ*efp* host. The recovered *truncated* protein was missing mass consistent with it lacking the last proline of the test motif and subsequent amino acids (calculated mass difference = 396.46 Da; observed mass difference = 395.63). The mass of this product was confirmed at higher resolution by measuring the doubly charged ion peaks and also by measuring the mass of the poly-proline region separately purified as a tryptic fragment (not shown). An unidentified contaminant was present in each sample with a mass ~210 Da heavier than the reporters.
(EPS)

S5 Fig EF-P abundance and distribution. EF-P was detected using Western blots with polyclonal antibodies. (**A**) EF-P levels were determined in normalized total protein samples from wild-type, Δ*efp::kan*, Δ*epmA::kan*, and Δ*epmB::kan* cells. (**B**) EF-P (top panel) and LepA (bottom panel) were detected in pooled sucrose gradient fractions from the *top*, 30S and 50S *subunits* region, *monosome* peak, and *polysome* region. Each pooled sample was precipitated with alcohol and resuspended in SDS-PAGE sample buffer for analysis. The band migrating above the EF-P band is not related to EF-P (detectable in knockout strains). The anti-LepA Western served as a control to evaluate protein content using a translation factor that also transiently associates with polysomes.
(EPS)

S6 Fig Small RNAs purified from wild-type and Δ*efp* sucrose gradients. RNA samples from wild-type and Δ*efp* gradients were electrophoresed to resolve small RNAs. The 23S, 16S, 5S rRNAs and tRNAs are labeled. The 5S distribution in the Δ*efp* gradient differs from the wild-type distribution with more of the total being present in the 50S position.
(EPS)

S7 Fig Removal of L9 does not compromise particle distributions. A comparison of 30S, 50S, and 70S peaks from wild-type and Δ*rplI::tet* cells (top row) an also cells that had the degradation system activated in cells with L9-cont and L9-deg (bottom row). The degradation system did not reduce 70S material and there was essentially no change in the relative particle abundances when L9 was absent or depleted. See **S8 Fig** for an example of a gradient depleted of L9. Error bars represent the standard deviations from three quantifications of lysates from two experimental repeats.
(EPS)

S8 Fig Depleting L9 in wild-type cells recapitulates Δ*rplI* defects. The L9 degradation system was activated in otherwise wild-type cells. (**A**) Westerns show the abundance of L9-cont and L9-deg before activation of the protease system and at the time of harvest. Sucrose gradients of the two lysates have similar peak intensities, but there is more immature RNA in the 30S peak of the L9-deg sample (*asterisk*). (**B**) RNA samples were prepared from polysomes recovered from either *top-down* or *bottom-up* fractionations of the same lysates. The inset shows RNAs from the recovered polysomes, immature 16S rRNA was not evident. RT-qPCR was used to quantify the lp16S (additional 115 5' nucleotides) and sp16S (additional 66 5' nucleotides) levels relative to total 16S. For comparison, the amount of immature 16S found in the top-down fractionated L9-cont gradient was set to 100%. Note that the fractionation method reversed the observed relative abundance. Error bars represent the standard deviation of four measurements from two experimental repeats.
(EPS)

S1 Table Genes sequenced in a fast-growing Δ*efp* escape mutant. Each was wild-type. Functional annotations derived from www.ecogene.org.
(DOCX)

S2 Table Cloned genes tested for multi-copy suppression of Δ*efp* sickness. ASKA library clones were transformed into Δ*efp* cells and evaluated for their ability to enhance the growth under different induction conditions (glucose = low, glycerol = moderate, IPTG = high) [70]. None improved the fitness. Annotations derived from www.ecogene.org.
(DOCX)
