# Supplementary material for: The Large Ribosomal Subunit Protein L9 Enables the Growth of EF-P Deficient Cells and Enhances Small Subunit Maturation
Source: PLoS One. 2015 Apr 16;10(4):e0120060. doi: 10.1371/journal.pone.0120060 (PMC4399890; doi:10.1371/journal.pone.0120060)
Supplement: S1 Table — Each was wild-type. Functional annotations derived from www.ecogene.org. (DOCX) [file pone.0120060.s009.docx]

| **Gene** | **Function/rationale** |
| --- | --- |
| *der* | ribosome biogenesis, mutation renders L9 dependence |
| *eno* | enolase, RNA degradosome component, in *relA* operon |
| *mazE* | antitoxin of MazF, in *relA* operon |
| *mazF* | RNase, toxin, in *relA* operon |
| *mazG* | NTPase, binds Era, in *relA* operon |
| *relA* | (p)ppGpp synthetase, stringent response regulator |
| *rlmD* | 23S rRNA m(5)U1939 methyltransferase, in *relA* operon |
| *rlmN* | 23S rRNA m(2)A2503, tRNA m(2)A37 methyltransferase, near *der* |
| *priB* | primosome component, in *rplI* operon |
| *recG* | DNA helicase, in *spoT* operon |
| *rplI* | L9, suppresses EF-P absence |
| *rpoZ* | RNAP omega subunit, in *spoT* operon |
| *rpsD* | S4, aminoglycoside resistance, miscoding (*ram*), or hyperaccuracy |
| *rpsE* | S5, aminoglycoside resistance or miscoding (*ram*) |
| *rpsL* | S12, aminoglycoside dependence (hyperaccuracy) |
| *rpsR* | S18, in *rplI* operon |
| *spoT* | (p)ppGpp synthetase/hydrolase, stringent response regulator |
| *trmH* | tRNA mG18 2'-O-methyltransfersase, in *spoT* operon |
