# Supplementary material for: The Large Ribosomal Subunit Protein L9 Enables the Growth of EF-P Deficient Cells and Enhances Small Subunit Maturation
Source: PLoS One. 2015 Apr 16;10(4):e0120060. doi: 10.1371/journal.pone.0120060 (PMC4399890; doi:10.1371/journal.pone.0120060)
Supplement: S2 Table — ASKA library clones were transformed into Δefp cells and evaluated for their ability to enhance the growth under different induction conditions (glucose = low, glycerol = moderate, IPTG = high) [70]. None improved the fitness. Annotations derived from www.ecogene.org. (DOCX) [file pone.0120060.s010.docx]

| **Gene** | **Function/rationale** |
| --- | --- |
| *deaD* | 50S subunit biogenesis |
| *infA* | translation initiation factor IF-1 |
| *infB* | translation initiation factor IF-2 |
| *infC* | translation initiation factor IF-3 |
| *ksgA* | 16S rRNA dimethyltransferase, mutation confers kasugamycin resistance |
| *rhlE* | RNA helicase in degradasome |
| *rimJ* | acetylates S5, 30S subunit biogenesis |
| *rluB* | 23S rRNA pseudouridine synthase |
| *rng* | RNase G, 16S processing |
| *rnpA* | RNase P, tRNA and 4.5S RNA processing |
| *rsgA* | 30S subunit biogenesis |
| *rsmC* | 16S rRNA methylase |
| *rsmE* | 16S rRNA methylase |
| *rsuA* | 16S rRNA pseudouridine synthase |
| *smpB* | tmRNA binding and ribosome rescue |
| *yhbC* | 30S subunit biogenesis |
| *yhbY* | 50S subunit biogenesis |
